# Enhanced alveolar ridge preservation with hyaluronic acid-enriched allografts: a comparative study of granular allografts with and without hyaluronic acid addition

**DOI:** 10.1186/s40729-024-00559-6

**Published:** 2024-10-09

**Authors:** Frank R. Kloss, Thomas Kau, Diana Heimes, Peer W. Kämmerer, Anita Kloss-Brandstätter

**Affiliations:** 1 Private Clinic for Oral, Maxillofacial and Plastic Facial Surgery, Kärntnerstraße 62, Lienz, 9900 Austria; 2 Department of Radiology, Landeskrankenhaus Villach, Nikolaigasse 43, Villach, 9500 Austria; 3 Department of Oral and Maxillofacial Surgery/Plastic Surgery, University of Mainz, Augustusplatz 2, Mainz, 55131 Germany; 4 Department of Engineering & IT, Carinthia University of Applied Sciences, Europastraße 4, Villach, 9524 Austria

**Keywords:** Socket preservation, Dental implants, Extraction sockets, Allogeneic bone material, Regenerative dentistry, maxgraft^®^, Hyaluronic acid

## Abstract

**Purpose:**

Ridge preservation is essential to restore alveolar ridge volume and to enhance esthetic and functional outcomes for dental implants. The addition of hyaluronic acid to allogeneic bone substitute materials might enhance these outcomes. This clinical study evaluated the efficacy of ridge preservation after tooth extraction using granular allografts with and without hyaluronic acid addition.

**Methods:**

In this retrospective study, 40 patients with compromised extraction sockets were enrolled. Among them, 19 received particulate allogeneic bone substitutes (Allo), 21 received allogeneic bone substitutes with hyaluronic acid (AlloHya). Vertical and horizontal graft stability, graft shrinkage rate, and bone mineral density were assessed using radiographic measurements on CBCT scans conducted before tooth extraction, directly after ridge preservation and after four months. Patients were followed up 12 months post-implantation.

**Results:**

Vertical height loss after 4 months was significantly greater in the Allo group (-0.82 ± 0.95 mm) compared to the AlloHya group (-0.19 ± 0.51 mm; *p* = 0.011). Graft shrinkage rate was 16.9 ± 11.5% (Allo) and 10.3 ± 7.7% (AlloHya) (*p* = 0.038). After four months, average bone density was significantly higher in the AlloHya compared to the Allo group (*p* = 0.004). Nearly all implants (39 out of 40) were classified as “Success” according to the ICOI scheme, with no differences in implant quality between the two study groups.

**Conclusions:**

Improved graft stability, reduced resorption, and increased bone density were observed in hyaluronic acid-enriched allografts compared to pure allografts. Adding hyaluronic acid to allogeneic bone grafts significantly enhanced outcomes in ridge preservation.

## Background

Alveolar ridge preservation is a vital procedure in dentistry and oral surgery, aimed at minimizing bone resorption and maintaining the alveolar ridge’s volume and morphology following tooth extraction [1]. Loss of alveolar bone after extraction can compromise the esthetic and functional outcomes of dental implant placement and prosthetic rehabilitation [2, 3]. Various techniques, including autografts, allografts, and xenografts, provide structural support and scaffolding for new bone formation, promoting osteogenesis and osteoconduction [4, 5]. Additionally, guided bone regeneration (GBR) techniques use barrier membranes to facilitate undisturbed bone regeneration, while growth factors like platelet-rich fibrin and bone morphogenetic proteins accelerate tissue regeneration and enhance bone formation [6].

Despite the advancements, there is no consensus on the optimal ridge preservation technique for different clinical scenarios [7, 8]. Studies have highlighted significant dimensional changes in hard and soft tissues post-extraction, with substantial reductions in horizontal and vertical dimensions impacting treatment planning and prosthetic rehabilitation [9, 10]. Current methods, although effective, vary in outcomes, necessitating further exploration of innovative materials and approaches [4, 11, 12]. Hyaluronic acid has emerged as a promising adjunct in alveolar ridge preservation due to its multifaceted benefits in tissue regeneration and wound healing [13]. Its viscoelastic properties help maintain hydration and structural integrity within the graft site, thereby reducing graft shrinkage and promoting vertical stability. Hyaluronic acid interacts with cell surface receptors to stimulate osteoblast activity and inhibit osteoclastogenesis, leading to enhanced bone density and improved integration of the graft material. Clinical studies have demonstrated that hyaluronic acid not only modulates inflammatory responses and enhances angiogenesis but also synergistically enhances the osteoconductive properties of graft materials, making it an effective component for improving outcomes in alveolar ridge preservation [14–17].

This study aimed to evaluate the clinical efficacy of ridge preservation using a hyaluronic acid-enriched allogeneic bone substitute material in patients with compromised extraction sockets. Over a 12-month period, the intervention group receiving the hyaluronic acid-enriched allograft was compared to a control group receiving an allogeneic bone substitute material without hyaluronic acid. The outcomes assessed included horizontal and vertical bone gain, volume stability, graft shrinkage, and bone mineral density. This comparison aimed to determine the potential benefits of adding hyaluronic acid to granular allografts in enhancing bone regeneration and soft tissue healing. This is the first clinical study to compare allogeneic bone substitutes with and without hyaluronic acid for alveolar ridge preservation.

## Methods

### Patients

A total of 40 patients presenting with decayed teeth necessitating extraction were enrolled in this retrospective study. Post-extraction, radiological evaluation using cone-beam computed tomography (CBCT) revealed defects in the buccal cortical plate, consistent with the clinical presentation of compromised extraction sockets. All patients needed single-implant treatments. After four months of healing time, every patient received one titanium implant per augmented region. All patients were fully informed about the surgical procedures and treatment alternatives. The minimum extraction socket defect type for inclusion was a Type-IV bone defect, as defined by Kim et al. 2021 [3]. Exclusion criteria consisted of a history of radiotherapy in the head and neck region, systemic disease that would contraindicate oral surgery, uncontrolled periodontal disease, bruxism, pregnancy, psychiatric problems, and/or use of medications known to alter bone healing.

Prior to surgery, patients were presented with two alternative procedures for ridge preservation (allogeneic bone grafts with or without hyaluronic acid). All patients were provided with standardized information sheets regarding allogeneic ridge preservation and dental implantation. In addition, patients were informed about the optional use of hyaluronic acid. The decision to opt for this addition was left entirely to the discretion of the patients, as no conclusive evidence regarding potential added benefits could be provided at the time of surgery. In order to reduce potential sources of bias, patients were selected for each of the two study groups so that they did not differ in demographic or anamnestic characteristics (Table 1). After screening the available clinical data, 19 patients with comparable demographic characteristics were allocated the “Allo” treatment group (no hyaluronic acid) and 21 patients were allocated the “AlloHya” treatment group (with hyaluronic acid).

**Table 1:** Demographic characteristics of the patient groups and implant success criteria, assessed 12 months post-implantation

	Allo group	AlloHya group	Statistical test	*p*-value
Gender	8 males; 11 females	13 males; 8 females	Chi-squared test	0.342
Age	48.68 ± 13.84	54.36 ± 10.23	t-test	0.146
Jaw	Maxilla: 8 Mandible: 5	Maxilla: 12 Mandible: 9	Chi-squared test	0.333
Locus	Incisors: 11 Premolars: 4 Molars: 4	Incisors: 12 Canine: 1 Premolars: 4 Molars: 4	Chi-squared test	0.814
Bleeding on probing	No: 16 Yes: 3	No: 20 Yes: 1	Fisher’s Exact test	0.331
Radiographic bone loss from initial bone level	< 2 mm: 10 2–4 mm: 8 > 4 mm: 1	< 2 mm: 13 2–4 mm: 8 > 4 mm: 0	Chi-squared test	0.523
Implant quality scale	Success: 18 Satisfactory: 1	Success: 21 Satisfactory: 0	Fisher’s Exact test	0.475

### Power calculations

For a statistical power calculation, we made the following assumptions: the average resorption rate for grafts materials ranged between 14.4% (SD 9.0) for allogeneic bone blocks [18] to 33.4% (SD 3.1) for xenogeneic granular graft materials [19]. Since the allogeneic material that we used was granular, we assumed that the resorption rate of the granular allogeneic material used in this study was similar to the resorption rate from xenogeneic granular graft materials. With these assumptions, we had statistical power of 100% to detect significant differences between the two study groups with 20 patients each and with a level of significance of 5%. Power calculations were performed with RStudio using the package “pwr”.

### Grafting materials

Two variants of allogeneic bone substitutes in granular form were utilized. On the one hand, granular maxgraft^®^ was used (study group “Allo”), and on the other hand, granular maxgraft^®^ + Hya (study group “AlloHya”) was applied (botiss biomaterials GmbH, Berlin, Germany).

maxgraft^®^ granules comprise allograft bone substitute derived from human donor bone, meticulously processed by the Cells + Tissuebank Austria using the specialized cleaning procedure known as the Allotec^®^ process. Offered in both cancellous and cortico-cancellous forms, maxgraft^®^ retains its natural bone structure and collagen composition. Serving as an effective scaffold, it facilitates the natural regeneration of bone tissue and holds the potential for complete assimilation into the patient’s own bone through remodeling [20].

maxgraft^®^ + Hya merges the allogeneic bone grafting material maxgraft^®^ with the hydrophilic properties of hyaluronic acid. Utilizing the notable liquid-binding capabilities of hyaluronate, maxgraft^®^ + Hya transforms into a cohesive and adhesive bone material upon hydration, commonly referred to as “sticky bone”. This transformation enhances application convenience by enabling straightforward uptake and precise delivery to the target site, thereby improving procedural efficiency. Prior to application, maxgraft^®^ + Hya requires rehydration. According to the manufacturer’s protocol, approximately 0.8 ml of saline solution is used per 1 ml of maxgraft^®^ + Hya.

### Surgical procedure

**Fig. 1:**
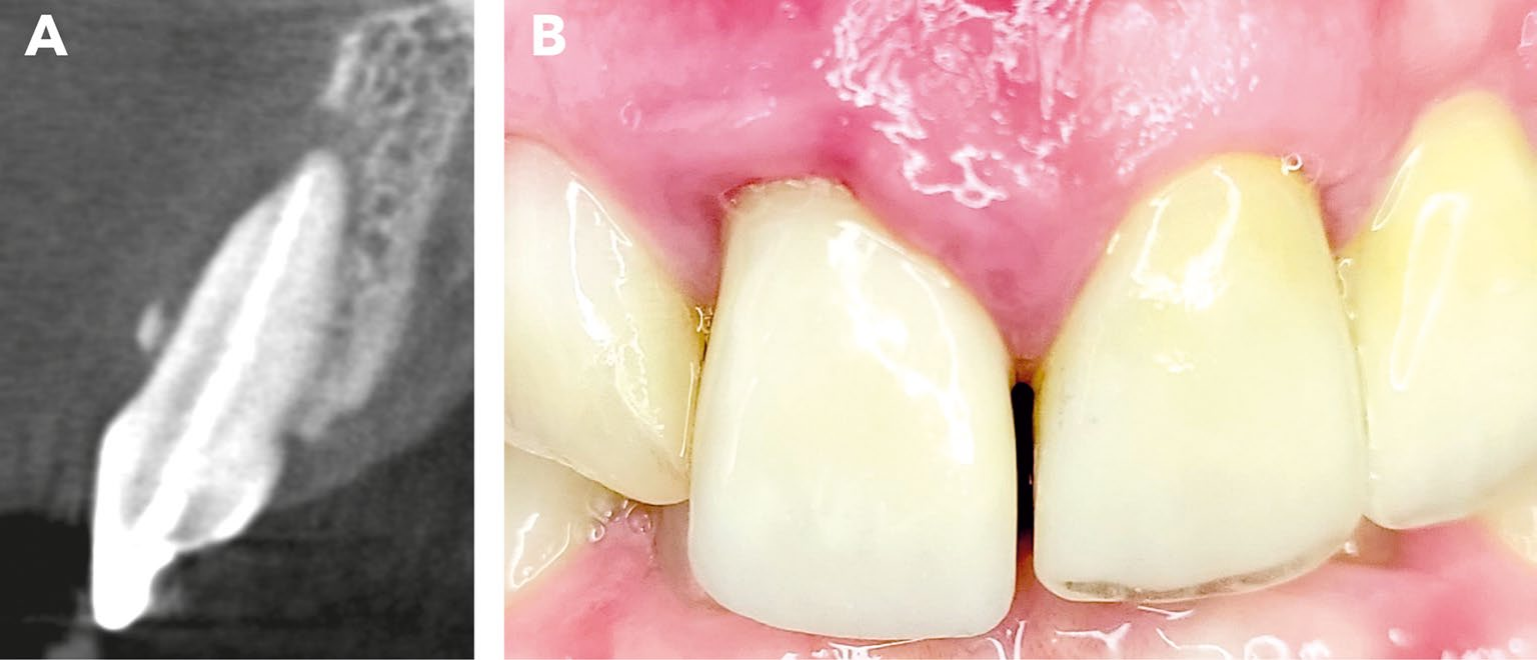
Initial clinical situation. The sectional image of the CBCT, along with the recession on tooth 11, reveals a class III defect according to Kim et al. [3]. The mucosa appears inflamed, and an extensive loss of the buccal alveolar wall is visible

**Fig. 2:**
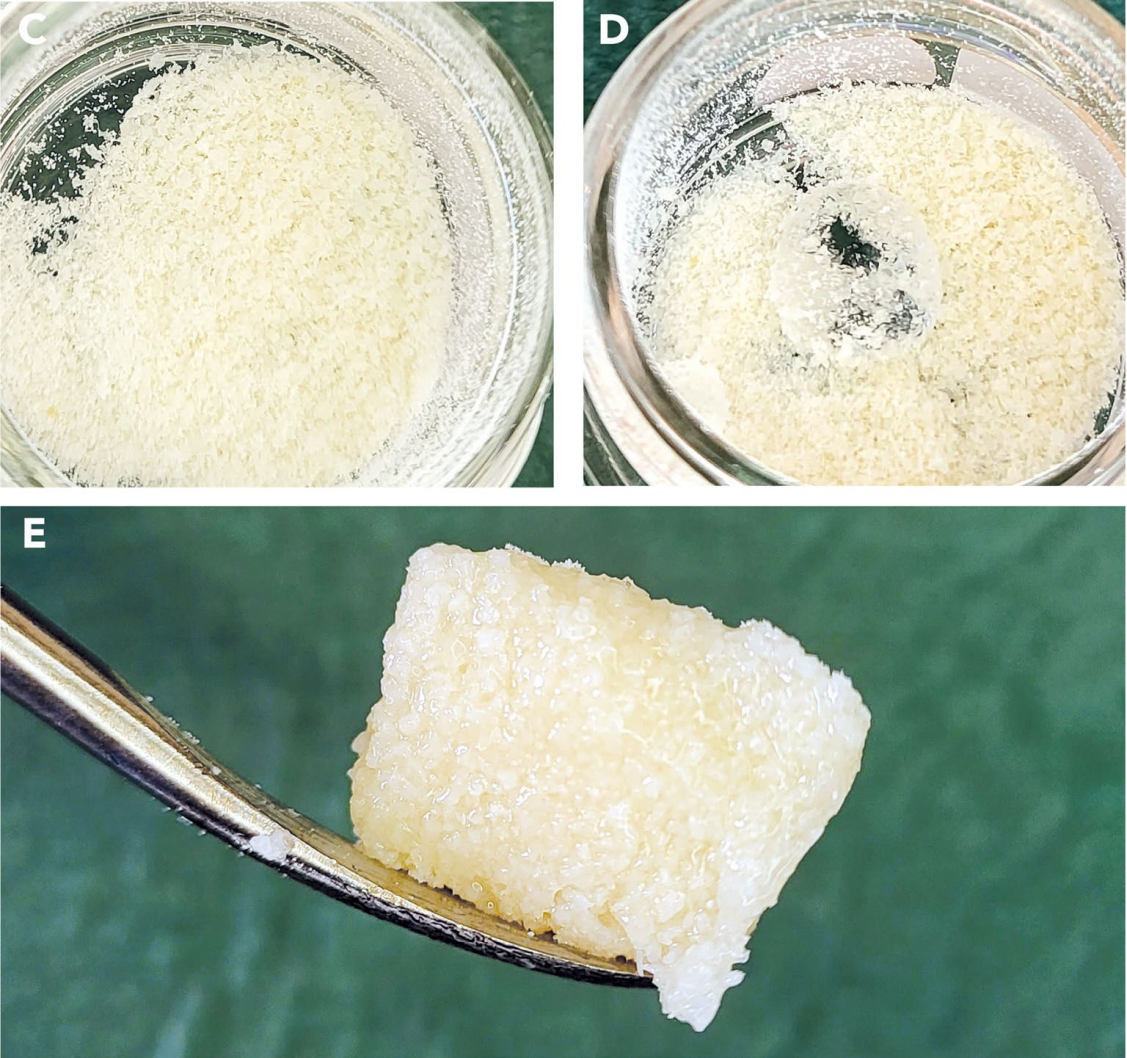
Hyaluronic acid enriched allograft. Figure C shows the allogenic granules mixed with powdered hyaluronic acid. Adding sterile NaCl solution (Figure D) produces a moldable mass known as “sticky bone” (Figure E)

**Fig. 3:**
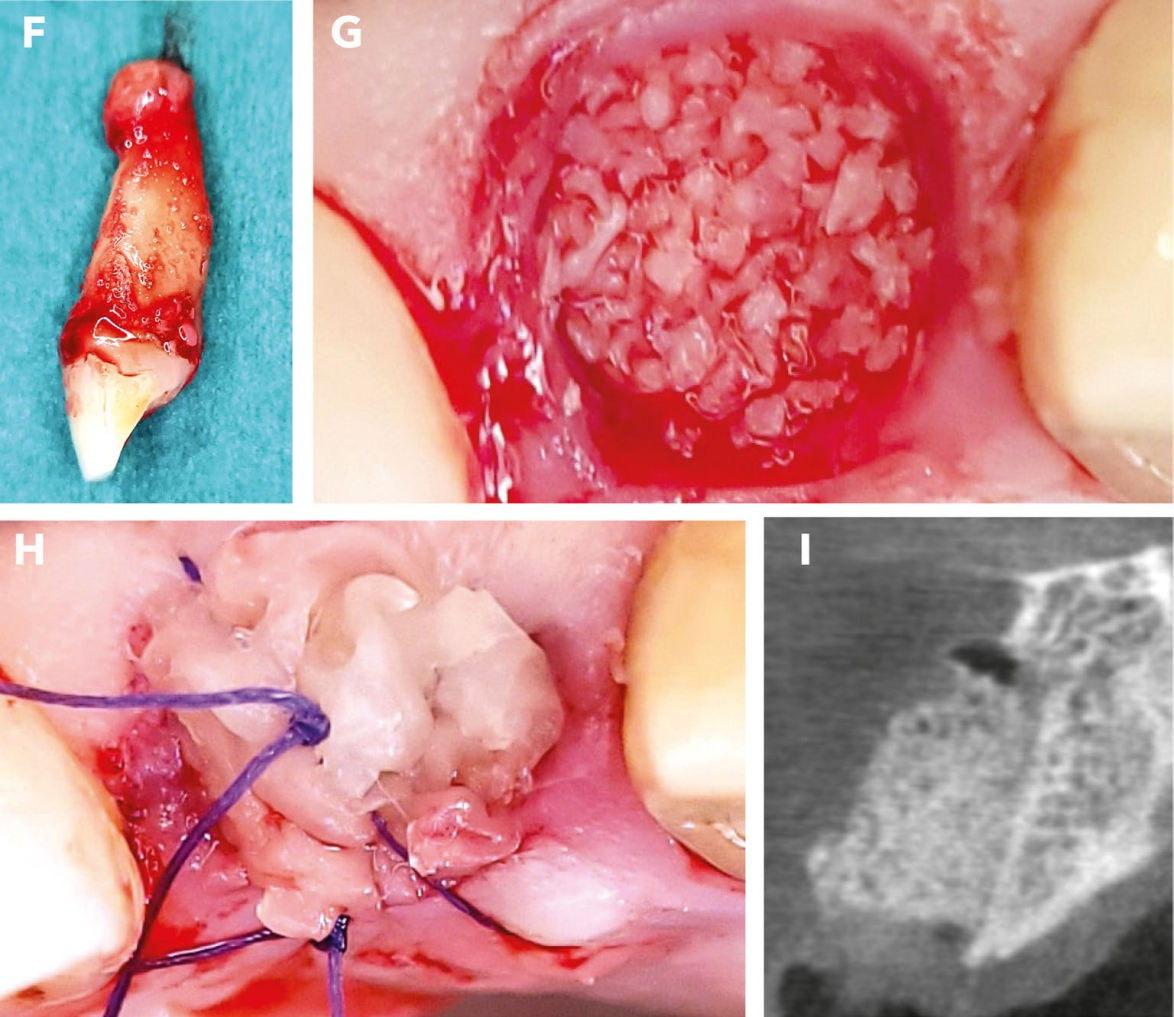
Alveolar ridge preservation. Tooth extraction (Figure F) revealed apical granulation. After mechanical cleaning, the socket was filled with allogenic bone substitute mixed with hyaluronic acid (Figure G). To optimize soft tissue healing, a PRF plug was placed in the socket and secured with a situational suture (Figure H). The postoperative control image (Figure I) shows the vestibular oversizing of the inserted material

Prior to extraction blood was drawn from the patient to prepare A-PRF (advanced platelet rich fibrin) following the protocol of Choukroun with a Mectron centrifuge [21, 22]. After atraumatic tooth extraction, the extraction site was thoroughly mechanically cleaned to remove any remaining debris and granulation tissue (Fig. 1). Once the site was prepared, allogeneic grafting material was rehydrated according to the manufacturer’s instructions with sterile saline and introduced into the socket to fill the void left by the extracted tooth (Fig. 2). The bone substitute was softly condensed. After placing the grafting material, A-PRF was positioned over the allograft and fixed with resorbable sutures (Fig. 3). Post-operative care instructions, including oral hygiene measures and dietary restrictions, were provided to the patient to ensure optimal healing and reduce the risk of complications. Routine post-operative care included administration of amoxicillin and clavulanic acid (625 mg, administered orally, three times a day for 4 days), ibuprofen (600 mg, administered orally, every 6 h as needed), and mouthwash (0.2% chlorhexidine, three times daily for 7 days).

.

**Fig. 4:**
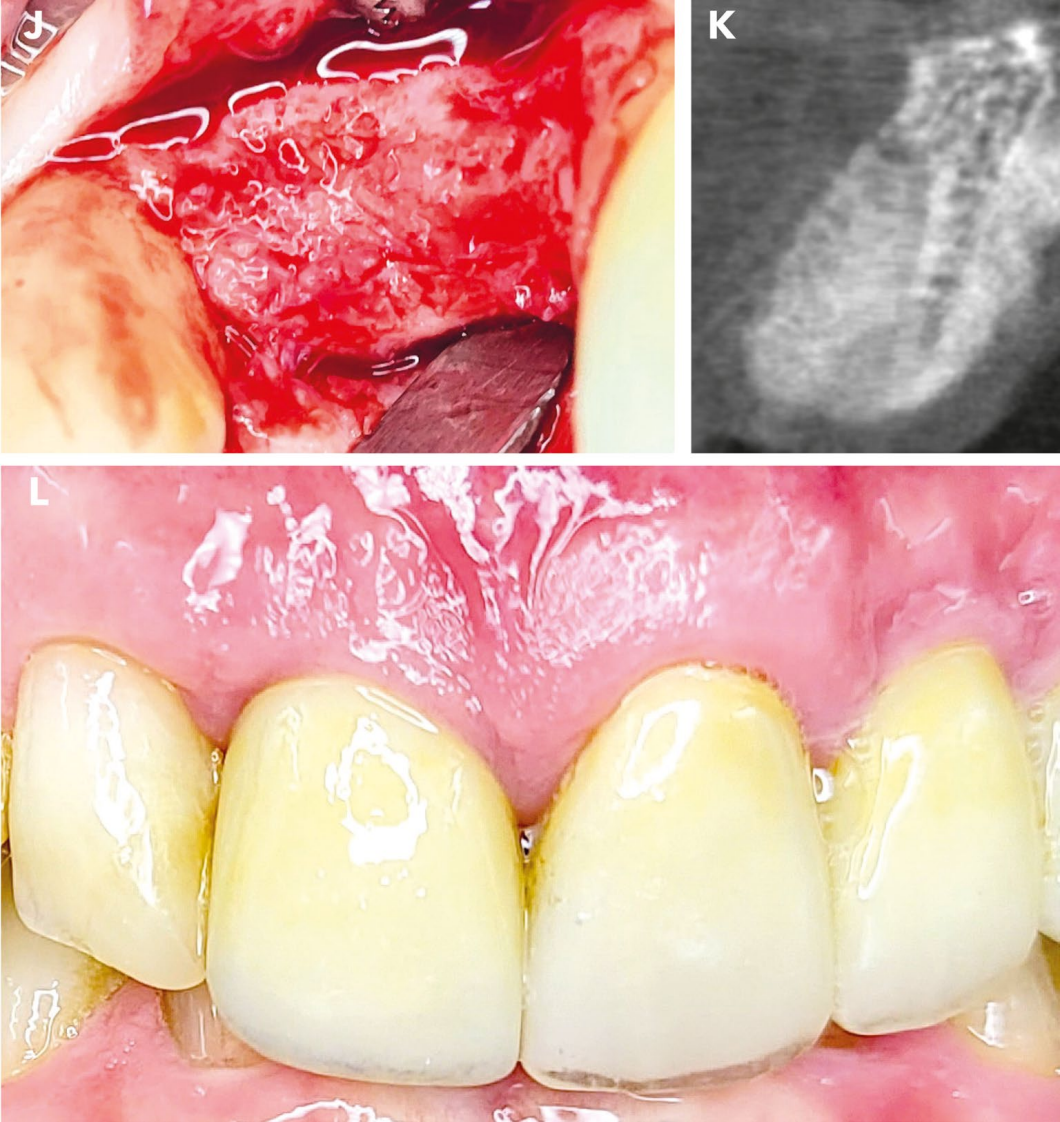
Final situation. After 4 months, when the surgical site was reopened (Figure J), a completely regenerated alveolar ridge was revealed. The alveolus showed complete radiological regeneration (Figure K), allowing for straightforward implant placement. Figure L illustrates the final prosthetic restoration with irritation-free mucosa conditions after one year

After four months of healing time, CBCT scans were done to investigate the osseous healing of the socket, and one titanium implant per augmented region was inserted (Fig. 4).

### Implant survival and success

Twelve months post-implantation, patients were recalled for a follow-up evaluation to assess implant success. The assessment was conducted using the guidelines from the International Congress of Oral Implantologists (ICOI) Pisa Consensus Conference on Implant Success, Survival, and Failure. Key parameters evaluated included pain, implant mobility, bleeding on probing, and radiographic bone loss relative to the initial bone level. These parameters were used to categorize the implants according to the ICOI implant quality scale [23].

### Radiographic analyses

**Fig. 5:**
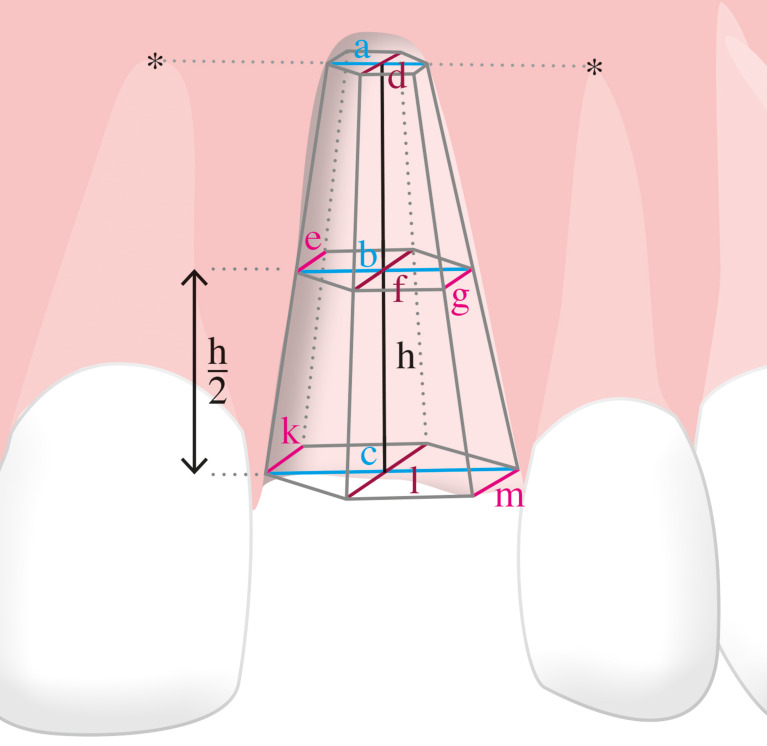
3D-Model for visualizing the mathematical approach of calculating the volume of the defect

Every patient was subjected to three-dimensional x-ray diagnostics (CBCT). In total three CBCTs were recorded for each patient, one before treatment, one directly after ridge preservation, and one after four months of healing before implantation. At each time point, the alveolar bone levels were measured in their height, width and depth at the cervical level, the middle height of the defect and at the apical level. An illustration of the measured regions is shown in Fig. 5.

The CBCT machine used to acquire all images was the Carestream CS 9300 (Carestream Health Inc., Onex Corporation, Rochester, New York, USA). The imaging parameters were set with a dose of 120 mGy.cm2, tube current of 18.66 mAs, and a voxel size of 90 μm x 90 μm x 90 μm. The selected field of view was 5 cm x 5 cm. Data from the scans were saved in the Digital Imaging and Communications in Medicine (DICOM) format and reconstructed with the Carestream implant planning software program.

All measurements were made on parasagittal sections perpendicular to the longitudinal axis of the adjacent teeth. The CBCT was oriented transversally through the teeth neighboring the defect so that the nerve canal of the tooth, which was mesial to the defect region, was visible. The nerve canal of the mesial tooth was defined as an anatomic reproducible landmark and a straight line was drawn through the middle of the defect region between the two neighboring teeth. The mesial tooth was used as a reference for apical and crestal bone levels. The distances were obtained using a software ruler. The same anatomic landmarks and distances were used for measurements on CBCT at the defined time intervals. The following measurements were taken (Fig. 5):

- Defect height (mm): distance between the apical and crestal bone level in the middle of the defect region; represented by line “h” in Fig. 5.
- Apical defect width (mm): distance between the apical root tips of the neighboring teeth; represented by line “a” in Fig. 5.
- Defect width in the middle zone (mm): distance between the roots of the neighboring teeth in the middle of the defect height; represented by line “b” in Fig. 5.
- Cervical defect width (mm): distance between the crestal bone levels of the neighboring teeth; represented by line “c” in Fig. 5.
- Apical defect depth (mm): distance between the labial/buccal and palatal edges of the jaw crest at the level of the apical tips of the neighboring teeth, but in the middle of the defect area; represented by line “d” in Fig. 5.
- Defect depth in the middle zone (mm): distances between the labial/buccal and palatal edges of the jaw crest at the level of the middle zone; represented by lines “e” (mesial region), “f” (central region) and “g” (distal region) in Fig. 5.
- Cervical defect depth (mm): distance between the labial/buccal and palatal edges of the jaw crest at the cervical level; represented by lines “k” (mesial region), “l” (central region) and “m” (distal region) in Fig. 5.

### Bone density

In order to estimate the bone density of the allograft, Hounsfield Units (HU) were used as a measurement of radiodensity. The integrated measurement module of a picture archiving and communication system software was used (PACS, DeepUnity Diagnost 1.1.1.1, DEDALUS). The regions of interest were defined in the graft’s crestal, middle and apical portions and adjusted to their size (5–20 mm^2^) at each level. In all cases, CBCT sections with low scattered radiation were chosen. The maximum area within the allograft was selected in such a way that the bony marginal structures of the alveolar process were not touched. Both the Hounsfield Units and the area of the measured region were recorded. For the purpose of determining the average bone density within a designated region of the CBCT scan, total HUs were divided by the area of the region measured. This provided the average radiodensity within that specific area.

### Mathematics and statistics

Based on the radiographic measurements, the graft volume was inferred as the sum of the volumes of two superimposed frustums of pyramids. The formula for obtaining the volume of one pyramid trunk is: $\:{V}_{pyramid\:trunk}=\frac{g}{3}\cdot\:\left(B+P+\sqrt{B\cdot\:P}\right)\:$, where “g*”* is the height of the truncated pyramid, “B” is the base area and “P” is the peak area.

The two pyramid trunks are depicted in Fig. 5. The formula for obtaining the volume of the entire defect was therefore:

$$\eqalign{& {V_{allograft}} = {h \over 6} \cdot \left( {{A_{crestal}} + {A_{middle}} + \sqrt {{A_{crestal}} \cdot {A_{middle}}} } \right) \cr & + {h \over 6} \cdot \left( {{A_{middle}} + {A_{apical}} + \sqrt {{A_{middle}} \cdot {A_{apical}}} } \right) \cr}$$

In the formula above, “h” denotes the distance between the apical and crestal bone level in the middle of the defect region. “A_crestal_” is the area of the cross-sectional surface of the alveolus at the crestal level. This was calculated from the width “c”, and the three horizontal, buccal-lingual depths “k” (mesial area), “l” (central area) and “m” (distal area). “A_middle_” is the area of the cross-sectional surface of the alveolus at the middle height of the defect. This was calculated from the width “b”, and the three horizontal, buccal-lingual depths “e” (mesial area), “f” (central area) and “g” (distal area). “A_apical_” is the area of the cross-sectional surface of the alveolus at the apical level. This was calculated from the width “a”, and the buccal-lingual depth “d”.

Statistical analyses were performed with IBM SPSS (version 27; International Business Machines Corp., Armonk, NY, USA).

Pearson’s chi-squared test was applied to sets of unpaired categorical data to evaluate the likelihood that any observed difference between the sets was due to chance. All metric variables were tested for normal distribution (Shapiro-Wilk test) and homogeneity of variance (Levene’s test) before parametric tests. An independent sample *t*-test was used when two separate sets of independent and identically distributed samples were obtained, and their population means were compared to each other. Multiple linear regression was used to try to explain an observed outcome variable (bone density) by several independent variables. The categorical variables were added to the model as factors. In the first model, all potential predictors were included. Then, all non-significant variables were removed from the multiple linear regression model in a stepwise manner.

Only two-sided significance tests were used. A probability of error of *p* ≤ 0.05 was chosen as the threshold value. An alpha adjustment for multiple testing was not performed. The results are therefore explorative and descriptive.

## Results

### Demographics

The demographic characteristics of the study population are summarized in Table 1. Gender was distributed evenly between the two study groups. There was no significant difference in the distribution of treated loci between the two study groups. The average age of the patients was 51.7 ± 12.2 years. The average healing time between ridge preservation and implantation was 4.1 ± 0.3 months.

### Implant survival and success

All patients were monitored for 12 months post-implantation. Throughout the healing period following ridge preservation, there were no indications of infection, wound dehiscence, graft exposure, or other postoperative complications. Allogeneic bone grafts were successfully integrated into the recipient sites by the time of implant placement. The grafted bone remained stable during drilling and implant placement in all patients, allowing for successful stabilization and restoration of all implants three months after placement. Each patient received a fixed implant-supported crown.

One year after implantation, no patients reported pain at the implant sites, and none of the 40 implants exhibited signs of mobility. Bleeding on probing was observed in 4 patients. Radiographic analysis revealed that the majority of implants (23 out of 40) showed no bone loss. Nearly all implants (39 out of 40) were classified as “Success” (group I) according to the ICOI scheme. No statistically significant differences were observed in implant quality or success criteria between the two study groups (Table 1).

### Vertical gain and graft stability

The remaining height of the alveolar bone before tooth extraction averaged 9.7 ± 2.5 mm (Table 2). After extraction and immediately following ridge preservation, the average height of the alveolar process at the extraction site was 10.1 ± 2.3 mm and did not differ between the two study groups. After four months of healing and therefore immediately before implant placement, the vertical height was the same in the two study arms. However, the vertical height loss after 4 months was significantly more pronounced in the Allo group than in the AlloHya group (*p* = 0.011).

**Table 2:** Vertical dimensional changes of the alveolus and its bony margins at three time points

Vertical dimensions (mm)	Allo group	AlloHya group	*p*-value
Height before tooth extraction	10.04 ± 2.68	9.34 ± 2.27	0.378
Height after ridge preservation	10.46 ± 2.39	9.81 ± 2.21	0.382
Height after 4 months	9.64 ± 2.32	9.63 ± 2.04	0.990
Vertical loss after 4 months	-0.82 ± 0.95	-0.19 ± 0.51	0.011*

### Horizontal gain and graft stability

To calculate the horizontal gain and loss rates, the cross-sectional areas were determined both at the crestal bone level (Table 3) and the mean height of the alveolus (Table 4). Before tooth extraction, the average cross-sectional area at the crestal bone level was 33.7 ± 19.2 mm^2^. Immediately after extraction and ridge preservation, the cross-sectional area at the crestal bone level amounted to 51.0 ± 24.3 mm^2^, and after four months the average values reached 44.5 ± 23.9 mm^2^. N statistically significant differences existed between Allo and AlloHya augmentations (Table 3). Horizontal bone loss at crestal bone level was --6.5 ± 4.6 mm^2^ and did not differ between the two groups (Table 3).

**Table 3:** Horizontal dimensional changes in the crestal area at three time points. The area of the alveolus and its bony margins was calculated from four distances

Horizontal dimension in crestal area (mm^2^)	Allo group	AlloHya group	*p*-value
Crestal area before tooth extraction	35.4 ± 18.54	32.16 ± 20.21	0.602
Crestal area after ridge preservation	50.63 ± 20.51	51.36 ± 27.87	0.926
Crestal area after 4 months	42.71 ± 19.4	46.09 ± 27.82	0.662
Horizontal loss after 4 months	-7.91 ± 5.03	-5.26 ± 3.86	0.068

**Table 4:** Horizontal dimensional changes in the central area of the tooth socket at three time points. The cross-sectional area of the alveolus and its bony margins was calculated from four distances

Horizontal dimension in central area (mm^2^)	Allo group	AlloHya group	*p*-value
Central area before tooth extraction	46.45 ± 36.49	47.98 ± 41.05	0.902
Central area after ridge preservation	52.39 ± 30.53	54.03 ± 40.58	0.887
Central area after 4 months	49.57 ± 30.87	50.79 ± 41.91	0.917
Horizontal loss after 4 months	-2.82 ± 5.96	-3.24 ± 4.57	0.806

At the mid-height of the socket, the average cross-sectional area before tooth extraction was 50.2 ± 36.6 mm^2^, immediately after ridge preservation the average cross-sectional area measured 53.3 ± 35.7 mm^2^ and after four months the average value came to 50.2 ± 36.6 mm^2^. Again, there was no difference between the two study groups (Table 4). The horizontal bone loss at the middle defect height was − 3.1 ± 5.2 mm^2^ and did not differ between Allo and AlloHya augmentations (Table 4).

### Remodeling

The three-dimensional volume of the defect area, i.e., the volume of the socket plus its bony margin, was 403.4 ± 321.2 mm^3^ before tooth extraction, 510.9 ± 363.8 mm^3^ immediately after extraction and subsequent ridge preservation and 449.9 ± 350.6 mm^3^ after 4 months of healing. There were no statistically significant differences in the volumes between Allo and AlloHya augmentations (Table 5). However, the augmentation volume shrank by an average of -80.7 ± 55.2 mm^3^ in ridge preservation without hyaluronic acid, while the volume of the augmentation with hyaluronic acid only decreased by an average of only − 43.2 ± 39.2 mm^3^ (*p* = 0.017). Therefore, the graft shrinkage rate was 16.9 ± 11.5% in ridge preservations without hyaluronic acid, while the graft shrinkage rate in ridge preservations with hyaluronic acid was only 10.3 ± 7.7% (*p* = 0.038).

**Table 5:** Volume changes of the alveolus and its bony margins at three time points

Volume dimensions (mm^3^)	Allo group	AlloHya group	*p*-value
Volume before tooth extraction	399 ± 261.41	407.43 ± 373.78	0.935
Volume after ridge preservation	510.36 ± 294.76	511.49 ± 424.17	0.992
Volume after 4 months	429.58 ± 269.1	468.27 ± 416.88	0.732
Volume loss after 4 months	-80.77 ± 55.26	-43.21 ± 39.25	0.017*
Graft shrinkage after 4 months	17.17 ± 10.96	10.61 ± 7.18	0.030*

### Bone density

Immediately after ridge preservation, the average bone density was 159.4 ± 66.1, and four months after ridge preservation, the average bone density was 176.5 ± 70.9. Bone density increased for both Allo and AlloHya augmentations over the four-month healing period. Immediately after ridge preservation, the average bone density was 121.31 ± 49.08 for augmentations without hyaluronic acid and 189.38 ± 64.38 for augmentations with hyaluronic acid (*p* < 0.01; Table 6). After a four-month healing period, the average bone density was 132.66 ± 48.85 for augmentations without hyaluronic acid and 211.03 ± 67.35 for augmentations with hyaluronic acid (*p* < 0.01; Table 6).

**Table 6:** Average bone density of the graft material directly after ridge preservation and after 4 months, prior to implantation

Average bone density	Allo group	AlloHya group	*p*-value
Bone density after ridge preservation	121.31 ± 49.08	189.38 ± 64.38	0.008**
Bone density after 4 months	132.66 ± 48.85	211.03 ± 67.35	0.004**

## Discussion

### Clinical relevance of and potential mechanisms behind the observations

The results of our study indicated that the addition of hyaluronic acid to allogeneic bone grafting material significantly improved outcomes in the preservation of compromised extraction sockets. Specifically, we observed enhanced graft stability, reduced graft resorption and increased bone density.

#### Enhanced Graft Stability

While there were no differences in the horizontal graft stability between allogeneic ridge preservations with and without hyaluronic acid, the vertical height loss after 4 months was significantly more pronounced in the Allo group (-0.82 mm) than in the AlloHya group (-0.19 mm). Hyaluronic acid is well-known for its ability to enhance tissue regeneration and wound healing [24–28]. Studies have suggested that hyaluronic acid promotes cellular adhesion and proliferation, which could contribute to the formation of a robust matrix within the graft site [29–32]. In an animal model, cross-linked hyaluronic acid significantly enhanced periodontal wound healing and regeneration in two-wall mandibular intrabony defects [17]. In the context of ridge preservation, the incorporation of hyaluronic acid might have facilitated better integration of the graft material with the surrounding tissues, leading to improved stability.

#### Reduced shrinkage rate

There were no statistically significant differences in the volumes between allogeneic ridge preservations with and without hyaluronic acid. However, the graft shrinkage rate was 16.9% in allogeneic ridge preservations without hyaluronic acid, while the graft shrinkage rate in allogeneic ridge preservations with hyaluronic acid was only 10.3%. Hyaluronic acid possesses unique viscoelastic properties, which could have mitigated the shrinkage of the graft material over time. By maintaining hydration levels and supporting the structural integrity of the graft, hyaluronic acid might have minimized the volume loss typically associated with bone graft resorption. Indeed, ridge preservation with a mixture of a bovine graft material and hyaluronic acid in an animal model prevented dimensional shrinkage and improved bone formation in compromised extraction sockets [14]. Furthermore, hyaluronic acid has been shown to modulate inflammatory responses [33, 34] and promote angiogenesis [35, 36], which could indirectly influence graft remodeling and reduce shrinkage. In our study, the graft shrinkage after 4 months amounted for ~ 17% in the Allo group and for ~ 11% in the AlloHya group. The reduced shrinkage rate linked to the addition of hyaluronic acid to allogeneic bone material was also observed when combining hyaluronic acid with xenogeneic bone material for alveolar ridge preservation [15]. Therefore, hyaluronic acid appears to limit the post-extractional alveolar bone resorption when either mixed with allogeneic or xenogeneic bone material.

#### Increased bone density

After a four-month healing period, the average bone density was 132.66 for allogeneic ridge preservations without hyaluronic acid and 211.03 for allogeneic ridge preservations with hyaluronic acid. Hyaluronic acid is involved in various signaling pathways that regulate osteogenesis [37] and bone remodeling [38]. By interacting with cell surface receptors such as CD44 and RHAMM [30, 35], hyaluronic acid can stimulate osteoblast activity and mineralization processes [39]. Additionally, hyaluronic acid has been shown to inhibit osteoclastogenesis and bone resorption, thereby preserving bone density [40, 41]. The addition of hyaluronic matrix to xenograft in maxillary sinus augmentation significantly increased bone surface density, suggesting enhanced bone quality [42]. The combination of hyaluronic acid with the allogeneic bone grafting material might have synergistically enhanced these osteogenic effects, resulting in higher bone density at the graft site. Differences in baseline density might be explained by the higher viscosity of hyaluronic acid in comparison to saline. However, considering the rapid turnover of hyaluronic acid in situ, this effect is unlikely to contribute to the differences after four months, in contrast even increasing the differences between grafting and four months later.

#### Potential synergistic effects

maxgraft^®^ is a widely used allogeneic bone grafting material known for its biocompatibility and osteoconductive properties [13, 43, 44]. The addition of hyaluronic acid could have complemented these characteristics by providing a biological component that promotes tissue regeneration and modulates the local microenvironment. Studies have suggested that hyaluronic acid can interact synergistically with other biomaterials, enhancing their therapeutic efficacy in various clinical applications [45, 46]. This was also demonstrated for platelet rich fibrin (PRF), which was found to be beneficial for ridge preservation surgeries, especially when combined with other graft materials [47].

#### Clinical evidence supporting hyaluronic acid supplementation

Previous studies investigating the use of hyaluronic acid in bone regeneration and dental procedures have reported favorable outcomes, supporting its efficacy as a therapeutic adjunct [24, 48–50]. A recent randomized clinical trial showed that adding hyaluronic acid to the coronally advanced flap procedure significantly improved complete root coverage and reduced post-operative swelling and discomfort in the treatment of gingival recessions [16]. Furthermore, adding cross-linked hyaluronic acid to demineralized bovine bone mineral during guided bone regeneration significantly improved bone quality and quantity compared to using bovine bone material alone [51]. Topical application of hyaluronic acid as an adjunctive treatment improved clinical outcomes in both non-surgical and surgical periodontal therapies [52].

In summary, the use of allogeneic bone substitutes combined with hyaluronic acid in ridge preservation offers advantages such as hydrophilic properties, enhanced cell attachment, reduced inflammation, periodontal regeneration, scaffold functionality, improved bone regeneration, bacteriostatic properties and scarless wound healing. By that, without changing the individual treatment protocol, an improved patient outcome can be achieved.

### Strength and limitations

Our study on ridge preservation boasts several significant strengths, including a robust sample size of 20 patients per group. The statistical power of 100% guarantees our ability to detect even subtle differences between the groups, adding to the reliability of our findings. Additionally, the introduction of a new biomaterial that has not been published before represents a novel contribution to the field. The homogeneity of our patient group further enhances the validity of our results, minimizing variability and potential confounding factors. However, our study is not without limitations. The method used to calculate bone density relies on grey values, which may not provide the most precise measurement. Moreover, the observation period of 12 months post-implantation may not capture the long-term outcomes and stability of the ridge preservation techniques evaluated.

Evaluating bone mineral density (BMD) at implant placement sites is crucial for ensuring sufficient primary stability. Computed tomography (CT) is widely acknowledged as the standard method for BMD assessment due to its consistent display of Hounsfield units (HUs). However, CT’s high radiation dosage restricts its use in dental diagnoses. A recent systematic review examined the relationship between cone-beam computed tomography (CBCT) gray values (GVs) and CT’s HUs in assessing BMD [53]. Converting CBCT’s linear attenuation coefficients into HUs requires applying a prediction equation model or conversion ratio to GVs. Despite limitations, both qualitative and quantitative analyses in the review revealed a positive correlation between CBCT’s GVs and CT’s HUs. Therefore, CBCT’s GVs can be utilized to quantitatively estimate bone density prior to implant-related procedures, supported by evidence indicating a positive correlation between CBCT’s GVs and CT’s HUs [53].

Although a positive influence on initial healing pattern caused by topical use of hyaluronic acid has been published [17], those parameters have not been evaluated in the present study but might be subject to further research.

The observation period of 12 months post-implantation can be considered less problematic given that all augmentation sites remained free of inflammation and dehiscence, and all implants demonstrated stability without any signs of peri-implantitis, bleeding on probing, or other complications.

## Conclusion

In conclusion, our study demonstrates that adding hyaluronic acid to allogeneic bone substitutes in ridge preservation leads to enhanced graft stability, reduced shrinkage rate, and increased bone density. These findings highlight the potential of hyaluronic acid to optimize ridge preservation procedures and promote successful implant integration.

## Data Availability

The datasets used and/or analyzed during the current study are available from the corresponding author on reasonable request.

## Abbreviations

BMD: Bone mineral density
CBCT: Cone beam computed tomography
CT: Computed tomography
GBR: Guided bone regeneration
GV: Gray value
HU: Hounsfield unit
ICOI: International Congress of Oral Implantologists
SD: Standard deviation
